# Supplementary material for: Serum 25-Hydroxyvitamin D Levels and Youth-Onset Type 2 Diabetes: A Two-Sample Mendelian Randomization Study
Source: Nutrients. 2023 Feb 17;15(4):1016. doi: 10.3390/nu15041016 (PMC9963923; doi:10.3390/nu15041016)

Table S10: MR-STROBE checklist

Item No.	Section	Checklist item	Page No.	Relevant text from manuscript
1	**TITLE and ABSTRACT**	Indicate Mendelian randomization (MR) as the study's design in the title and/or the abstract if that is a main purpose of the study	1-2	Vitamin D and youth-onset type 2 diabetes: a Mendelian randomization study
	**INTRODUCTION**			
2	**Background**	Explain the scientific background and rationale for the reported study. What is the exposure? Is a potential causal relationship between exposure and outcome plausible? Justify why MR is a helpful method to address the study question	2-3	Due to the potential anti-inflammatory and immune-modulating properties of vitamin D, its role in childhood diabetes has been extensively studied in the case of the autoimmune type 1 diabetes[6], but data on youth-onset T2D are sparse. Case-control studies have shown that adolescents with insulin resistance have lower levels of 25 hydroxyvitamin D (25OHD), the bi-omarker of Vitamin D in humans [7], but this does not necessarily apply to pediatric patients with prediabetes or T2D[8,9]. One of the approaches which has been widely used to study causality be-tween vitamin D and T2D in adults[12] is Mendelian randomization (MR).<br>Under specific assumptions, MR uses single nucleotide polymorphisms (SNPs) as instruments for a modifiable exposure to study causal effects of this exposure on a disease-outcome[20].
3	**Objectives**	State specific objectives clearly, including pre-specified causal hypotheses (if any). State that MR is a method that, under specific assumptions, intends to estimate causal effects	3	In this study, we aimed to test whether genetically decreased 25OHD levels are causally associated with risk of youth-onset T2D in a mixed-ancestry and in ethnic-specific cohorts using MR. To do this, we leveraged data from the largest available European and ancestry-specific GWAS on 25OHD lev-els[21,22] and on the only available to date multi-ethnic GWAS on pediatric T2D[23].
	**METHODS**			
4	**Study design and data sources**	Present key elements of the study design early in the article. Consider including a table listing sources of data for all phases of the study. For each data source contributing to the analysis, describe the following:	4	See Figure 1
	a)	Setting: Describe the study design and the underlying population, if possible. Describe the setting, locations, and	3-4	In order to test causal effects of 25OHD on risk of pediatric T2D within the MR framework, we first

		relevant dates, including periods of recruitment, exposure, follow-up, and data collection, when available.		obtained conditionally independent SNPs associated with 25OHD in a large European GWAS meta-analysis of the SUNLIGHT consortium with UK BIOBANK totaling 443,734 individuals[21]. In this GWAS, 25OHD levels were measured using the Diasorin assay, and the mean 25OHD was 70 nmol/L. The effect of these SNPs on risk of pediatric T2D were sought in the multi-ethnic PRODIGY GWAS cohort (n=3,006 youth cases [mean age 15.1 years] and 6,061 adult controls) [23]. The 3,006 cases of the PRODIGY combined 449 youth with T2D from the TODAY study with >2,000 youth with T2D from a TODAY ancillary genetics study, and 468 youth with T2D from SEARCH for Diabetes in Youth. The adult controls were retrieved from the T2D-GENES study.<br><br>We also undertook ethnic-specific analyses by extracting effects of the 25OHD SNPs in the three ethnic sub-cohorts of PRODIGY (Non-Hispanic Whites, n=664 cases/1,434 controls;, African-Americans, n= 1,068 cases/1,068 controls; and Hispanics, n= 1,274 cases/3,559 controls).
	b)	Participants: Give the eligibility criteria, and the sources and methods of selection of participants. Report the sample size, and whether any power or sample size calculations were carried out prior to the main analysis	3-4	See answer to the point above
	c)	Describe measurement, quality control and selection of genetic variants	5-6	In order to conduct any MR study, the SNPs used as instrumental variables (IVs) of an exposure must satisfy three main assumptions. The first as-sumption (relevance assumption) requires that these SNPs should be strongly associated with the exposure, in this case, the 25OHD levels. This is ensured by using SNPs associated with 25OHD at a genome-wide significant level (p-value <5 x 10-8). We also calculated the F-statistic for our set of 25OHD SNPs, as a metric of the strength of our MR instruments.
	d)	For each exposure, outcome, and other relevant variables, describe methods of assessment and diagnostic criteria for diseases	5	Descriptives of the GWAS populations can be found in the respective GWAS publications[21,23].
	e)	Provide details of ethics committee approval and participant informed consent, if relevant		Not applicable.
5	**Assumptions**	Explicitly state the three core IV assumptions for the main analysis (relevance, independence and exclusion restriction) as well assumptions for any additional or sensitivity analysis	5	In order to conduct any MR study, the SNPs used as instrumental variables (IVs) of an exposure must satisfy three main assumptions. The first as-sumption (relevance assumption) requires that these SNPs should be strongly associated with the exposure, in

				this case, the 25OHD levels. This is ensured by using SNPs associated with 25OHD at a genome-wide significant level (p-value <5 x 10-8). We also calculated the F-statistic for our set of 25OHD SNPs, as a metric of the strength of our MR instruments.<br><br>The second assumption (independence assumption) requires that the SNPs used as MR instruments should not be associated with confounders of the association between the exposure and outcome. For instance, in this study, a possible confounder can be the body mass index (BMI), since obesity is as-sociated with lower 25OHD levels[26] and truncal adiposity is and estab-lished risk factor for both adult and pediatric T2D[1]. Another confounder of the association between 25OHD and T2D risk is ethnicity, with Afri-can-Americans presenting lower 25OHD levels and increased risk of T2D[27]. We undertook multiple sensitivity analyses with different sets of 25OHD SNPs to ensure that our MR results are not biased by the above confounders. Additionally, we performed a multivariable MR analysis considering both 25OHD and pediatric BMI as exposures. The third MR assumption (exclusion restriction assumption) requires that the SNP-IVs are associated with the outcome (here pediatric T2D) solely via the exposure (here 25OHD levels). Pleiotropy refers to a scenario where this assumption is vi-olated. In order to test for presence of pleiotropy, we conducted sensitivity analyses applying various pleiotropy-robust MR methods, each one with its own assumptions.
6	**Statistical methods: main analysis**	Describe statistical methods and statistics used		
	a)	Describe how quantitative variables were handled in the analyses (i.e., scale, units, model)	7	We computed the power in our main MR analyses, using the full set of 25OHD SNPs in the mixed-ancestry PRODIGY or the ethnic-specific PRODIGY sub-cohorts using an established MR power calculation method [42]. Specifically, we computed the MR odds ratio (OR) for pediatric T2D for which we obtained a power of 80%, setting the alpha level at 0.05, using the variance explained of 25OHD by its respective genetic instruments, and the sample sizes of the entire PRODIGY and its ethnic sub-cohorts.
	b)	Describe how genetic variants were handled in the analyses and, if applicable, how their weights were selected	7	The TwoSampleMR R package (version 0.5.6) [41], and its default parameters (LD-clumping r2=0.001) was used to select 25OHD SNPs, harmonize them between the exposure and outcome GWAS, and

			calculate the various MR estimates [IVW, weighted median, MR-Egger and weighted mode] of our main and sensitivity analyses. Random effects IVW was used, given the evidence of heterogeneity in our main analyses. Scatter plots and forest plots to visualize the MR estimates were generated using the TwoSampleMR R package. We used the MVMR R package for our multivariable MR analy-sis[42]. Our MR-PRESSO analyses were performed using the MR-PRESSO R package (version 1.0)[37].
c)	Describe the MR estimator (e.g. two-stage least squares, Wald ratio) and related statistics. Detail the included covariates and, in case of two-sample MR, whether the same covariate set was used for adjustment in the two samples	6	Using the PhenoScanner database[28], we filtered the MR instruments for 25OHD for SNPs with previously reported GWAS association with con-founders of the 25OHD-pediatric T2D association. This approach has been described in detail in previous MR studies by our group[29,30]. Specifically, we conducted sensitivity MR analyses excluding 25OHD SNPs associated at a genome-wide level with BMI, body composition traits, and adult-onset T2D, since family history of adult-onset T2D is a strong risk factor for youth-onset T2D.<br><br>To further account for effects of BMI on our MR estimates, we conducted a multivariable MR (MVMR) analysis[31]. To do this, we queried the effects of 25OHD SNPs on childhood BMI from a large European GWAS by the EGG consortium (a meta-analysis of 14 studies consisting of 5,530 cases, and 8,318 controls) [32].<br><br>Finally, to further account for confounding due to ancestry, we conducted a sensitivity analysis selecting three directly matching rare SNPs (rs14355701 in TINK, rs116950775 in KIAA1644/LDOC1L and rs111955953 in FTMT) from an African-American 25OHD GWAS on 697 individuals[22], which were combined to a common SNP in GC (rs4588) identified in a recent larger GWAS meta-analysis on 2,602 African American adults from the Southern Community Cohort Study and 6,934 African- or Caribbean-ancestry adults from the UK Biobank[33]. Effects (betas) of these SNPs on 25OHD were ex-tracted from the above African 25OHD GWAS and their effects on pediatric T2D were identified in the African-American subset of PRODIGY.
d)	Explain how missing data were addressed	4	For 25OHD-related variants not directly present in the ethnic-specific PRODIGY GWAS, we selected proxy SNPs (LD r 2> 0.7) using the LDproxy function in ldlink[24] in matching populations from the 1000 genomes phase 3 panel.

	e)	If applicable, indicate how multiple testing was addressed		Not applicable
7	**Assessment of assumptions**	Describe any methods or prior knowledge used to assess the assumptions or justify their validity	5	In order to conduct any MR study, the SNPs used as instrumental variables (IVs) of an exposure must satisfy three main assumptions. The first assumption (relevance assumption) requires that these SNPs should be strongly associated with the exposure, in this case, the 25OHD levels. This is ensured by using SNPs associated with 25OHD at a genome-wide significant level (p-value $<5 \times 10^{-8}$). We also calculated the F-statistic for our set of 25OHD SNPs, as a metric of the strength of our MR instruments.<br>The second assumption (independence assumption) requires that the SNPs used as MR instruments should not be associated with confounders of the association between the exposure and outcome. For instance, in this study, a possible confounder can be the body mass index (BMI), since obesity is associated with lower 25OHD levels[26] and truncal adiposity is and established risk factor for both adult and pediatric T2D[1]. Another confounder of the association between 25OHD and T2D risk is ethnicity, with African-Americans presenting lower 25OHD levels and increased risk of T2D[27]. We undertook multiple sensitivity analyses with different sets of 25OHD SNPs to ensure that our MR results are not biased by the above confounders. Additionally, we performed a multivariable MR analysis considering both 25OHD and pediatric BMI as exposures. The third MR assumption (exclusion restriction assumption) requires that the SNP-IVs are associated with the outcome (here pediatric T2D) solely via the exposure (here 25OHD levels). Pleiotropy refers to a scenario where this assumption is violated. In order to test for presence of pleiotropy, we conducted sensitivity analyses applying various pleiotropy-robust MR methods, each one with its own assumptions.
8	**Sensitivity analyses and additional analyses**	Describe any sensitivity analyses or additional analyses performed (e.g. comparison of effect estimates from different approaches, independent replication, bias analytic techniques, validation of instruments, simulations)	4-7	See answers to points 5,6,7 of the checklist
9	**Software and pre-registration**			

	a)	Name statistical software and package(s), including version and settings used	7	The TwoSampleMR R package (version 0.5.6) [41], and its default parameters (LD-clumping r2=0.001) was used to select 25OHD SNPs, harmonize them between the exposure and outcome GWAS, and calculate the various MR estimates [IVW, weighted median, MR-Egger and weighted mode] of our main and sensitivity analyses. Random effects IVW was used, given the evidence of heterogeneity in our main analyses. Scatter plots and forest plots to visualize the MR estimates were generated using the TwoSampleMR R package. We used the MVMR R package for our multivariable MR analysis[42]. Our MR-PRESSO analyses were performed using the MR-PRESSO R package (version 1.0)[37].
	b)	State whether the study protocol and details were pre-registered (as well as when and where)		Not applicable
	**RESULTS**			
10	**Descriptive data**			
	a)	Report the numbers of individuals at each stage of included studies and reasons for exclusion. Consider use of a flow diagram	4	Figure 1
	b)	Report summary statistics for phenotypic exposure(s), outcome(s), and other relevant variables (e.g. means, SDs, proportions)	3	In order to test causal effects of 25OHD on risk of pediatric T2D within the MR framework, we first obtained conditionally independent SNPs associated with 25OHD in a large European GWAS meta-analysis of the SUNLIGHT consortium with UK BIOBANK totaling 443,734 individuals[21]. In this GWAS, 25OHD levels were measured using the Diasorin assay, and the mean 25OHD was 70 nmol/L (SD 34.7 nmol/L). The effect of these SNPs on risk of pediatric T2D were sought in the multi-ethnic PRODIGY GWAS cohort (n=3,006 youth cases [mean age 15.1 years] and 6,061 adult controls) [23]. The 3,006 cases of the PRODIGY combined 449 youth with T2D from the TODAY study with >2,000 youth with T2D from a TODAY ancillary genetics study, and 468 youth with T2D from SEARCH for Diabetes in Youth. The adult controls were retrieved from the T2D-GENES study.
	c)	If the data sources include meta-analyses of previous studies, provide the assessments of heterogeneity across these studies		See answer to point above

	d)	For two-sample MR:<br>i. Provide justification of the similarity of the genetic variant-exposure associations between the exposure and outcome samples<br>ii. Provide information on the number of individuals who overlap between the exposure and outcome studies		To our knowledge, there is no overlap between exposure data and outcome data
11	**Main results**			
	a)	Report the associations between genetic variant and exposure, and between genetic variant and outcome, preferably on an interpretable scale	8	As shown in Table 1, our MR estimates remained largely consistent and close to the null across different MR methods, with the exception of the weighted median MR showing a marginal effect of a SD increase in log-transformed 25OHD on pediatric T2D risk in the mixed-ancestry cohort (OR pediatric T2D 1.09, 95% CI 1.00-1.18, P = 0.049) and in Hispanics (OR 1.13, 95% CI 1.02-1.26, P=0.019).
	b)	Report MR estimates of the relationship between exposure and outcome, and the measures of uncertainty from the MR analysis, on an interpretable scale, such as odds ratio or relative risk per SD difference		See point above
	c)	If relevant, consider translating estimates of relative risk into absolute risk for a meaningful time period		Not applicable
	d)	Consider plots to visualize results (e.g. forest plot, scatterplot of associations between genetic variants and outcome versus between genetic variants and exposure)		See Supplemental Figure S1-S4
12	**Assessment of assumptions**			
	a)	Report the assessment of the validity of the assumptions	9	As shown in Table 1, the intercept of the MR-Egger provided no evidence of unbalanced horizontal pleiotropy in any of the MR studies, but there was significant heterogeneity among the MR instruments in all MR analyses except the one in African-Americans. All 25OHD SNPs used as instruments in our MR analyses had an F-statistic >10 (the average F-statistic was 234) (Tables S1-S4). The Steiger directionality test indicated that the correct causal direction was "TRUE" in all our main MR analyses, confirming that the assumption that altered 25OHD levels cause pediatric T2D (and not the inverse) was valid.

	b)	Report any additional statistics (e.g., assessments of heterogeneity across genetic variants, such as I^2, Q statistic or E-value)	9	See point above
13	**Sensitivity analyses and additional analyses**			
	a)	Report any sensitivity analyses to assess the robustness of the main results to violations of the assumptions	9	See point above. After using 4 SNPs in known vitamin D genes, as well as 4 African-American 25OHD SNPs, the consistent estimates were generated (Supplemental Tables 5-7)
	b)	Report results from other sensitivity analyses or additional analyses	9	As demonstrated in Tables S5 and S6, the results of our MR sensitivity analyses using four SNPs in known vitamin D genes (explaining 1.03% of the variance in 25OHD), as well as the four African-American 25OHD SNPs, did not indicate any causal effect of 25OHD on pediatric T2D either. Our sensitivity MR analysis excluding SNPs with effects on BMI, body composition traits or adult T2D in the PhenoScanner database yielded similar results as those of the main analysis (Table S7) with the exception of a significant estimate of the weighted median analyses in the mixed-ancestry and the Hispanic PRODIGY cohorts. Finally, the results of the MVMR testing for mediating effects of pediatric BMI were equally non-significant (Table S8).
	c)	Report any assessment of direction of causal relationship (e.g., bidirectional MR)	9	Steiger MR directionality test
	d)	When relevant, report and compare with estimates from non-MR analyses		Not applicable
	e)	Consider additional plots to visualize results (e.g., leave-one-out analyses)		Not applicable
	**DISCUSSION**			
14	**Key results**	Summarize key results with reference to study objectives	9	First paragraph of the Discussion
15	**Limitations**	Discuss limitations of the study, taking into account the validity of the IV assumptions, other sources of potential bias, and imprecision. Discuss both direction and magnitude of any potential bias and any efforts to address them	10	Our MR study has a few considerable limitations. One of these is that we did not study causal effects of altered levels of the active form of vitamin D [1,25 di-hydroxyvitamin D or 1,25(OH)2D] due to lack of available large GWAS on this form of vitamin D

				allowing to extract strong instruments for 1,25(OH)2D. Another limitation of our MR study is that the 25OHD SNPs which were used as instruments explain only up to 3.1% of variance in 25OHD levels in the Non-Hispanic White analysis, and only 2.5% of its variance in the other MR analyses. This, combined to the limited sample size of the PRODIGY GWAS, and in particular of its ancestry-specific sub-GWAS, has affected the power of our study to identify small and moderate effects of 25OHD on pediatric T2D risk. We elected to use as instruments in our main mixed-ancestry and an-cestry-specific MR analyses 25OHD SNPs identified in a large European vitamin D GWAS. This could have introduced bias due to the fact that effects of these SNPs on 25OHD levels in non-Europeans can differ substantially from those identified in a European GWAS. Nevertheless, there is no available 25OHD GWAS in Hispanics, and the results of our sensitivity analysis using African-American specific 25OHD SNPs were similar to those of the analysis using European 25OHD SNPs. Future large ances-try-informed GWAS on 25OHD and pediatric T2D will enable better power to interrogate their associations in various ethnic populations, including populations not studied in this work, using MR. Finally, the two-sample MR design of our study did not allow to undertake a stratified MR analysis to assess non-linear effects of 25OHD levels, since there are no available 25OHD data in PRODIGY participants. As such, effects of extremely low or high 25OHD levels on risk of pediatric T2D cannot be excluded.
16	**Interpretation**			
	a)	Meaning: Give a cautious overall interpretation of results in the context of their limitations and in comparison with other studies	10	This MR study does not support the use of vitamin D supplements to prevent T2D in youth of different ethnic backgrounds, but we cannot exclude small to moderate causal effects.
	b)	Mechanism: Discuss underlying biological mechanisms that could drive a potential causal relationship between the investigated exposure and the outcome, and whether the gene-environment equivalence assumption is reasonable. Use causal language carefully, clarifying that IV estimates may provide causal effects only under certain assumptions	9-10	Our findings suggest that the low 25OHD levels observed in adolescents with insulin resistance in an observational study[7], are not likely to be causal for their altered glucose metabolism, but they are rather driven by confounders, such as obesity. On the other hand, adolescents with obesity tend to be less physically active are spend less time outdoors, and as such can be less ex-posed to sunlight and may have decreased skin synthesis of vitamin D[9]. Moreover, they often have poor diet and suboptimal vitamin D

				intake[26]. Another possible explanation for the observed associations between low 25OHD levels and risk of pediatric T2D is ancestry. In this respect, a study [8] showed that, although vitamin D deficiency or insufficiency appeared to affect a substantial proportion of youth with T2D, and particularly non-Europeans, their prevalence was similar to that in youth without dia-betes. Taken together, all the above mechanisms support the presence of various possible confounders in the association between vitamin D and pe-diatric T2D..
	c)	Clinical relevance: Discuss whether the results have clinical or public policy relevance, and to what extent they inform effect sizes of possible interventions	10	See Conclusion section
17	**Generalizability**	Discuss the generalizability of the study results (a) to other populations, (b) across other exposure periods/timings, and (c) across other levels of exposure	10	See paragraph on limitations
	**OTHER INFORMATION**			
18	**Funding**	Describe sources of funding and the role of funders in the present study and, if applicable, sources of funding for the databases and original study or studies on which the present study is based	11	See relative section
19	**Data and data sharing**	Provide the data used to perform all analyses or report where and how the data can be accessed, and reference these sources in the article. Provide the statistical code needed to reproduce the results in the article, or report whether the code is publicly accessible and if so, where	11	R scripts used to generate the results of this study are available upon request to the corresponding author. Summary-level results of all GWAS used in this study are publicly available through GWAS catalog.
20	**Conflicts of Interest**	All authors should declare all potential conflicts of interest	11	The authors declare no conflict of interest.

Figure S1: Scatter plot and forest plot of the main MR analysis in the multi-ethnic PRODIGY cohort.

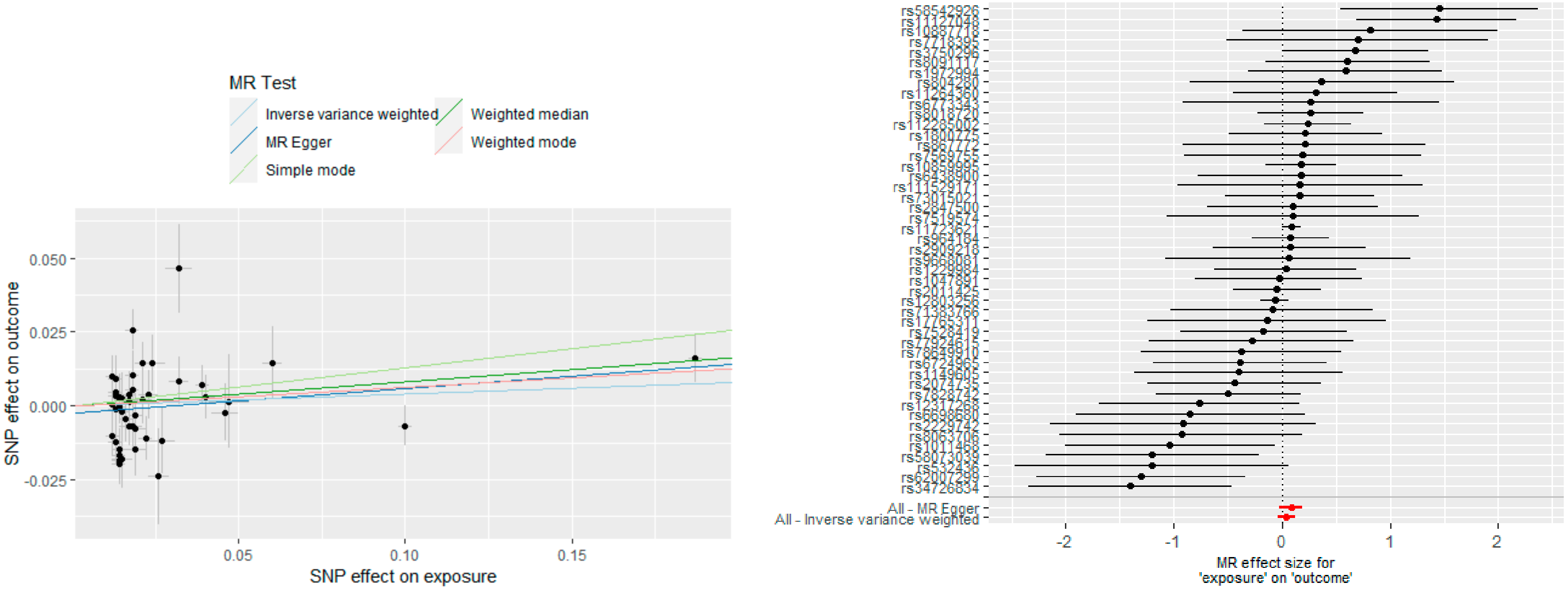

Figure S2: Scatter plot and forest plot of the main MR analysis in Non-Hispanic White PRODIGY sub-cohort.

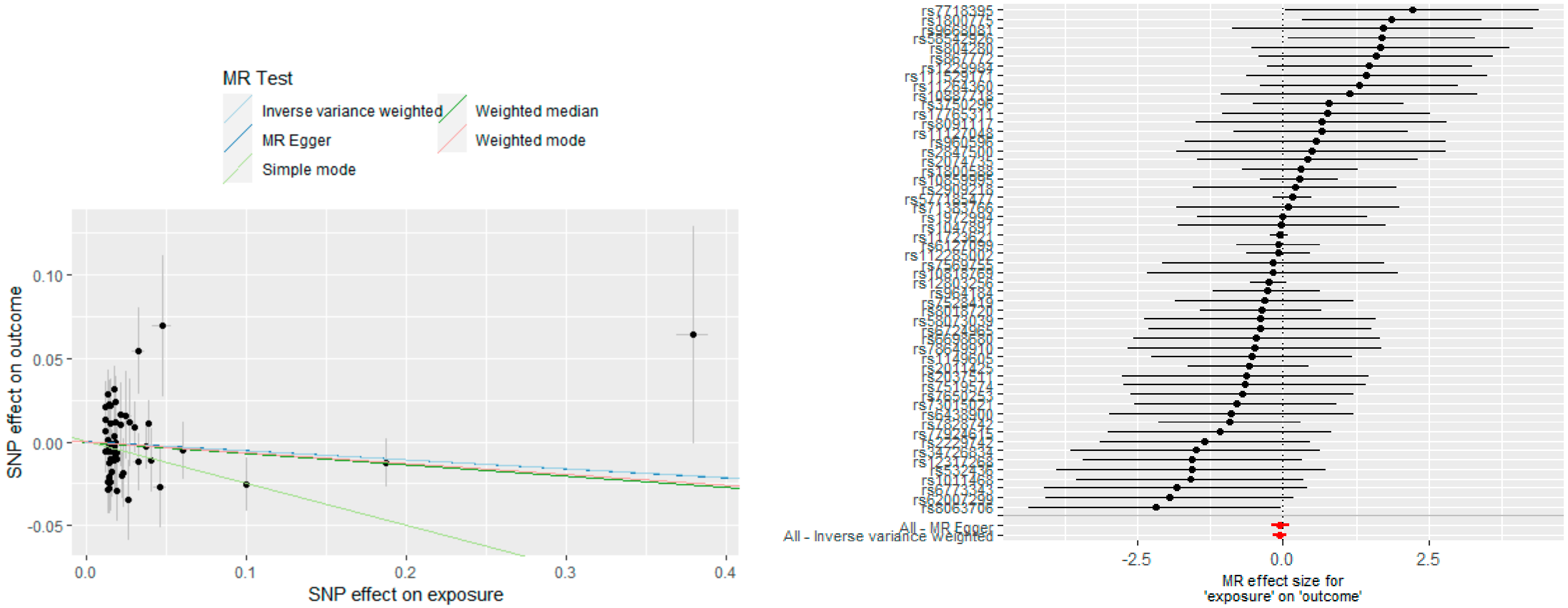

Figure S3: Scatter plot and forest plot of the main MR analysis in the African-American PRODIGY sub-cohort.

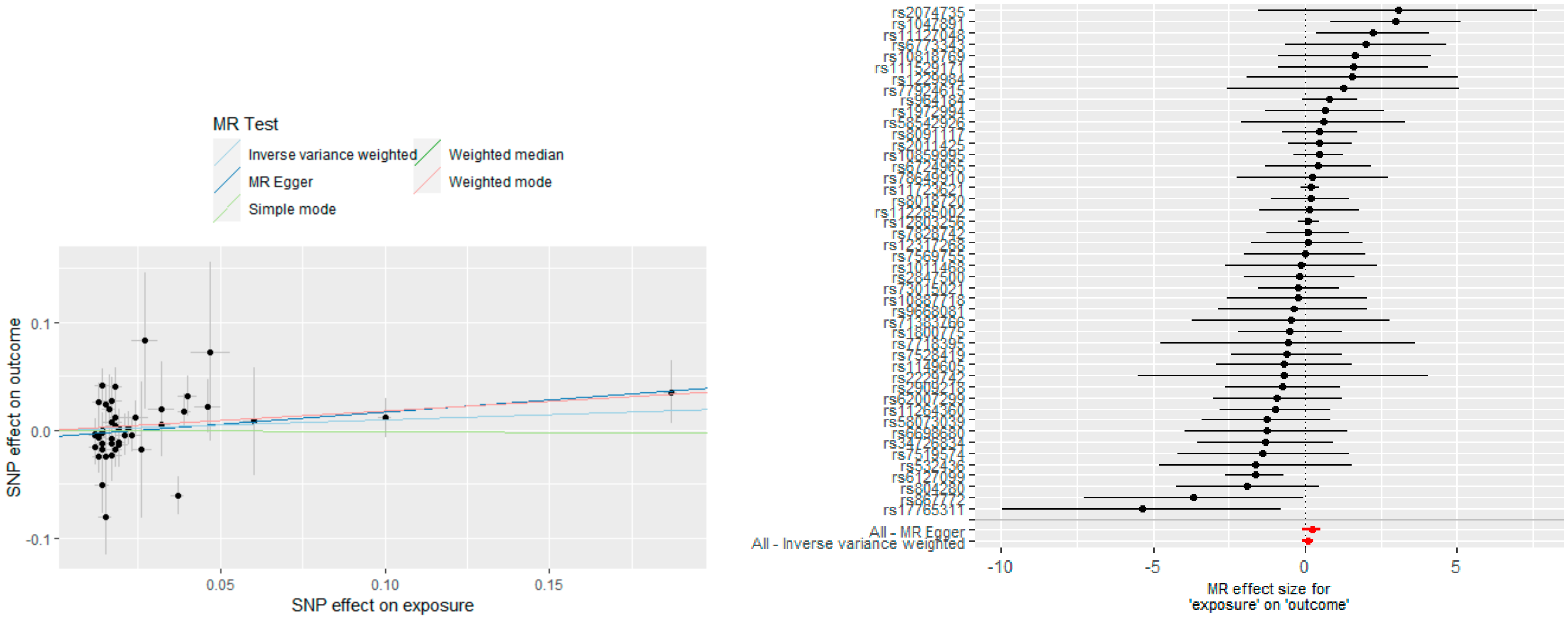

Figure S4: Scatter plot and forest plot of the main MR analysis in the Hispanic PRODIGY sub-cohort**.**

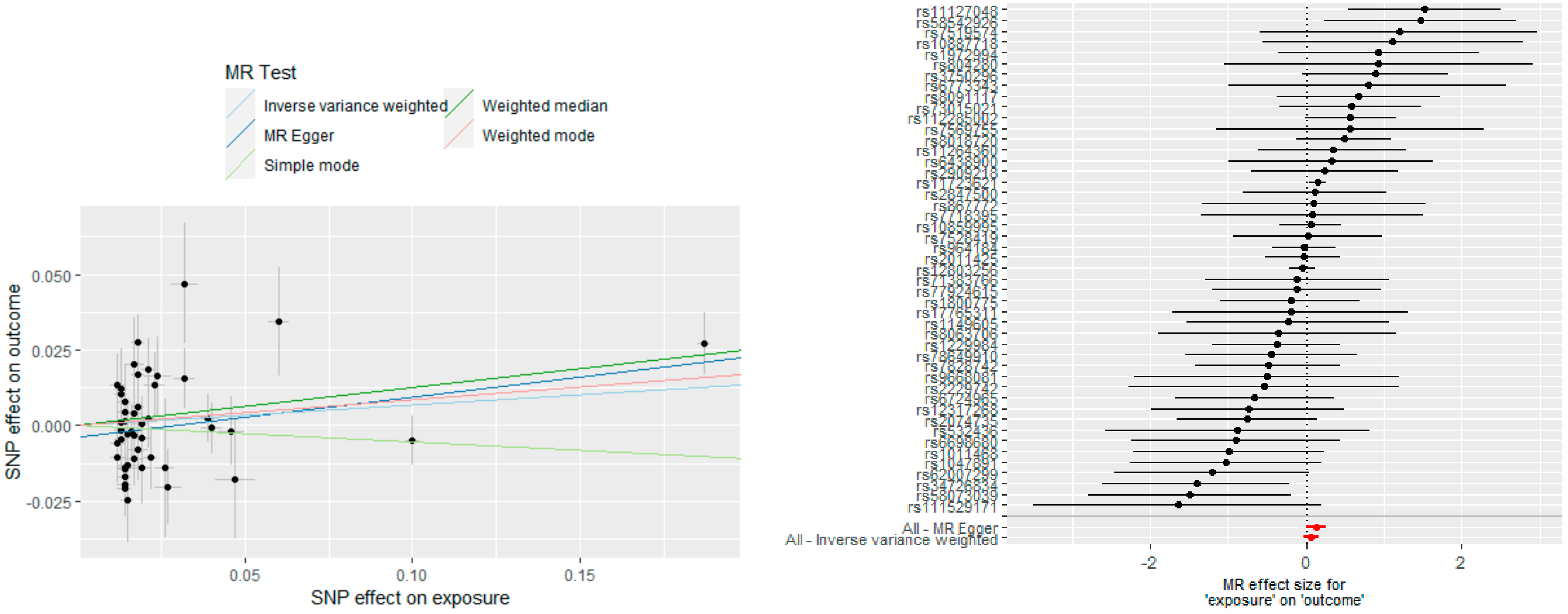

Supplement: Supplementary file 1 [file nutrients-15-01016-s001.zip › nutrients-2190361-supplementary.pdf]
